# Photosynthesis and food security: the evolving story of C_4_ rice

**DOI:** 10.1007/s11120-023-01014-0

**Published:** 2023-04-17

**Authors:** Robert Furbank, Steven Kelly, Susanne von Caemmerer

**Affiliations:** 1 Division of Plant Science, Centre of Excellence for Translational Photosynthesis, Research School of Biology, Australian National University, Acton, ACT Australia; 2 Department of Plant Sciences, University of Oxford, Oxford, UK

**Keywords:** C_4_ photosynthesis, CO_2_ concentrating mechanism, Crop yield, Rice

## Abstract

Traditional “Green Revolution” cereal breeding strategies to improve yield are now reaching a plateau in our principal global food crop rice. Photosynthesis has now become a major target of international consortia to increase yield potential. Synthetic biology is being used across multiple large projects to improve photosynthetic efficiency. This review follows the genesis and progress of one of the first of these consortia projects, now in its 13th year; the Bill and Melinda Gates funded C_4_ Rice Project. This project seeks to install the biochemical and anatomical attributes necessary to support C_4_ photosynthesis in the C_3_ crop rice. Here we address the advances made thus far in installing the biochemical pathway and some of the key targets yet to be reached.

## Photosynthesis and food security

The global population has now passed 8 billion and is predicted to reach more than 9 billion by the year 2040. Much has been written about our lack of capacity to feed this burgeoning population, with declining arable land areas, climate change, extreme weather events and stagnating progress in yields of our major cereal crops (UNICEF 2022; Furbank et al. 2020). While statistics such as the need for a 70% increase in food production by 2050 are sobering, the focus on the future can result in the lack of political will to address the issue with immediacy (WorldBank 2022). It has been suggested that the global food crisis, unlike COVID 19, is a silent pandemic, not causing the level of global response required to resolve the problem. Recent events comprising extreme and cataclysmic weather, rapidly rising fuel costs, supply chain issues, war and socioeconomic unrest have reminded us that the equation where food demand outstrips supply is near at hand. Indeed, we now appear to be revisiting the food crisis of 2008 which saw rapidly escalating food prices following the exhaustion of dwindling global grain reserves, resulting in famine and social unrest (FAO 2009).

While the causes of declining global food security are manifest, it is now widely accepted that the strategies used to achieve the huge “Green Revolution” gains in cereal grain productivity and land use gains (40–60% in wheat; Vietmeyer 2011; Stevenson et al. 2013) have largely been exhausted. Yield gains from reducing investment of fixed carbon into unproductive biomass such as stems via introduction of dwarfing genes, breeding for grain number and harvest index (the proportion of crop biomass comprising harvestable grain) appear to have plateaued (Furbank et al. 2020). As grain yield is a product of this “harvest index” and final crop biomass, increasing biomass through increases in photosynthetic efficiency have been identified as the next critical breeding target. Rice breeding has led the way for the global push to improve crop photosynthesis by utilizing an engineering approach based on basic scientific research, physiological knowledge and modelling.

## Rice is limited by photosynthate supply

**Fig. 1:**
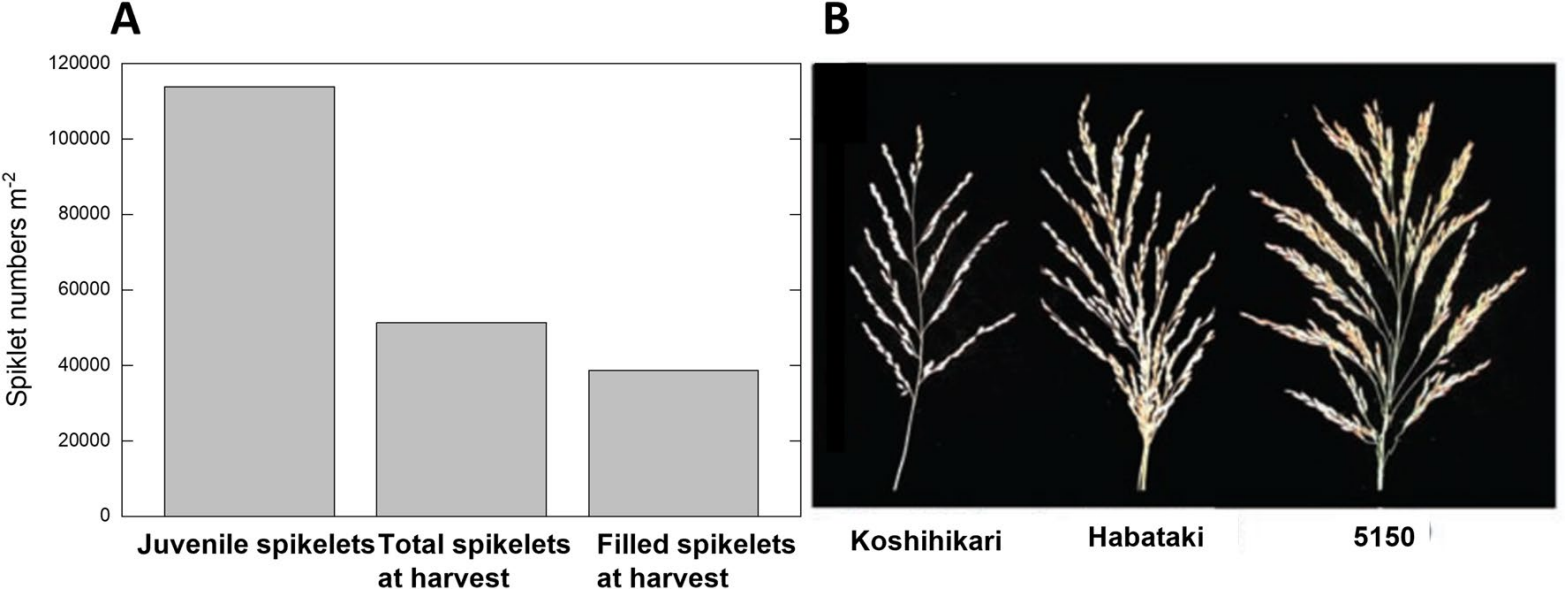
Spikelet numbers for indica rice variety IR72 averaged across 1997 and 1999 growing season data at the International Rice Research Station Los Banos Philippines (IRRI). From Sheehy et al (2001). 1000 grain weight in IR72 was 24 g in both seasons. Figure 1B shows typical panicles from Koshikari and Habataki; parents used to generate a QTL mapping population for grain number by Ashikari et al (2005) wherein a polymorphism in the cytokinin oxidase gene was identified as a major causative QTL for grain number. Line 5150 is a high yielding variety from China shown to have a deletion at the cytokinin oxidase locus resulting in a null mutation. These authors also phenocopied this large panicle architecture by producing a transgenic rice where cytokinin oxidase expression was suppressed

As early as the 1990’s, while debate as to the relationship between photosynthetic performance and yield was ongoing in other crops, in rice it was becoming clear that rice yields were limited by the supply of photosynthate (Sheehy et al. 2001). In the decade leading up to this realization, intensive breeding for a “new plant type” had been ongoing at the International Rice Research Institute (IRRI) in the Philippines, in China and elsewhere with less than encouraging results in some cases. Rice bred for high spikelet number actually produced less than 50% of the predicted yield, with less than half the juvenile spikelet’s reaching maturity as filled grain (Fig. 1A and Sheehy et al. 2001). Highly successful breeding for improvements in panicle spikelet number could in some cases even reduced the number of final filled grain, presumably by diluting the available photosynthate to the point that fertility was reduced and abortion increased. This was taken as clear evidence that high yield potential rice is “source limited” i.e. limited by the provision of photoassimilates to support grain filling. Similar source limitation is not seen in other crops such as wheat, where there is significant genetic variation in photosynthetic capacity across historic collections and elite material (Silva-Pérez et al. 2019), and photosynthetic supply has increased in concert with more gradual gains in sink demand (Fischer et al. 1998). We now know that in a number of rice breeding programs, that the step change in spikelet number was achieved due to a mutation of an enzyme of the cytokinin catabolism, cytokinin oxidase (Ashikari et al. 2005; Fig. 1B), resulting in sink demand outstripping carbon supply from leaves. Thus, in rice the demand and potential for large yield gains is present, and efforts to fill these additional spikelets has become a priority activity in rice breeding programs around the world.

## Redesigning rice photosynthesis to increase yield: the genesis of the C_4_ rice program

**Fig. 2:**
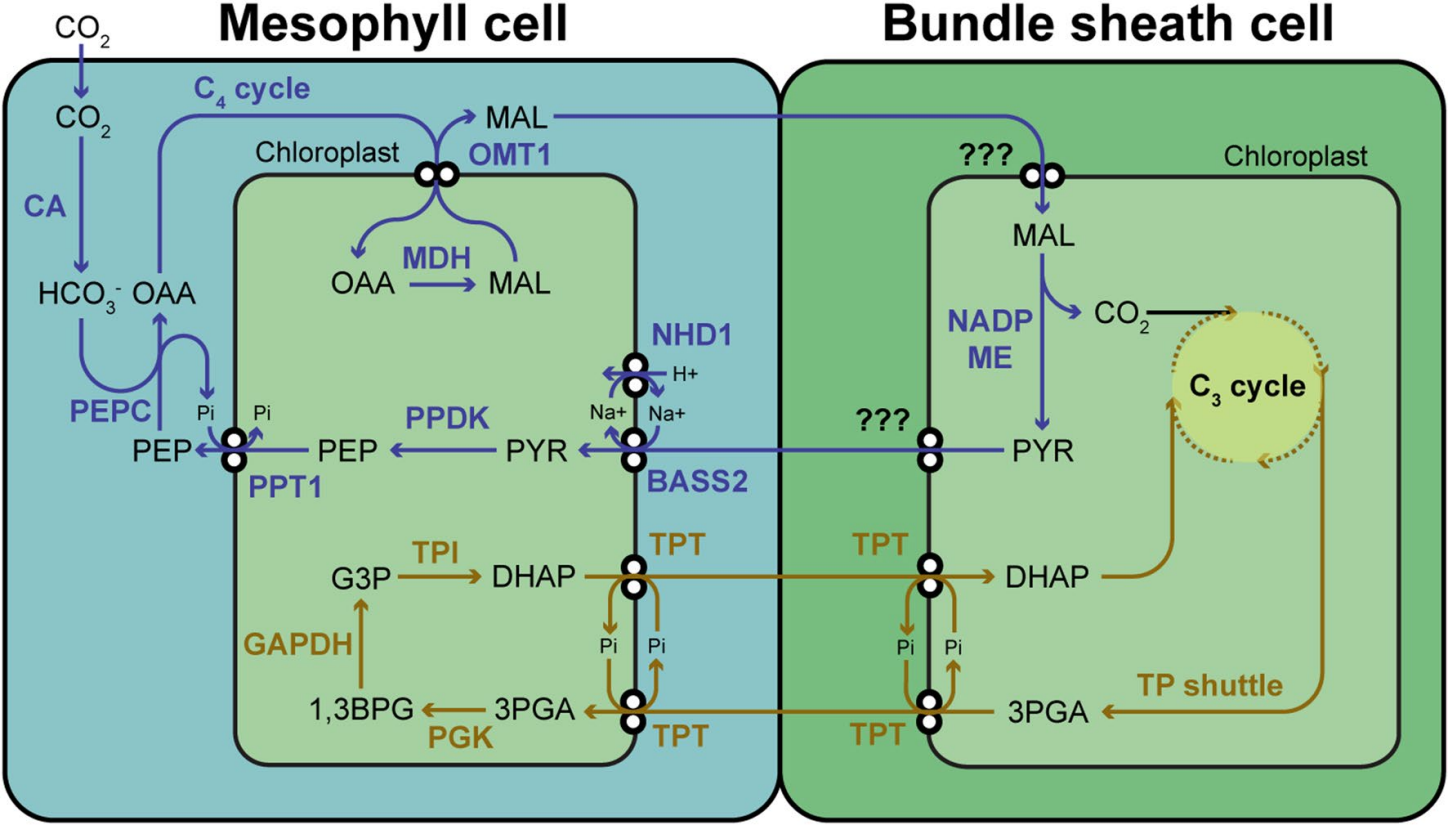
Schematic of enzymes and transporters included in the construction of C4 rice (in blue). These enzymes included carbonic anhydrase (CA), PEP carboxylase (PEPC), malate dehydrogenase (MDH), NADP-malic enzyme (NADP-ME), and pyruvate phosphate dikinase (PPDK). The transporters included are oxaloacetate/malate translocator (OMT1), pyruvate/sodium symporter (BASS2), Sodium/proton antiporter (NDH1), and PEP/phosphate translocator (PTT1). In bundle sheath chloroplasts it is unknown what is counter exchanged for malate or how pyruvate is exported. Also shown is the possible triose phosphate shuttle of the C3 cycle between bundle sheath and mesophyll cells. PYR pyruvate, PEP phospho*enol*pyruvate, OAA oxaloacetate, MAL malate, 3PGA 3-phosphoglycerate, 1,3BPG 1,3-bisphosphoglycerate, G3P glycerate-3-phosphate, DHAP dihydroxyacetone phosphate. TPT triose-phosphate/phosphate translocator, PGK phosphoglycerate kinase, GAPDH glyceraldehyde-3-phosphate dehydrogenase, TPI triosephosphate isomerase

John Sheehy, a crop modeler working with physiological breeders at IRRI, recognized that photosynthetic improvement was key to boosting rice yields and brought together an international group of plant biologists to IRRI at the end of 1999 to brainstorm the problem. While a multitude of ideas were discussed (Sheehy et al. 2000), Sheehy’s favourite option was a bold and ambitious plan to use genetic technologies to introduce into rice the entire C_4_ photosynthetic pathway, including anatomical specialization. He had calculated from modelling that this alone, among the options discussed, could improve radiation use efficiency to the level required to produce a 50% yield boost; the improvement required to meet projected demand in 2050 (Sheehy et al. 2000). In most species the C_4_ pathway is a complex combination of both biochemical and morphological specialisation, which provides an elevation of the CO_2_ concentration at the site of Rubisco in the bundle sheath. The C_4_ cycle, often called a biochemical CO_2_ pump, fixes CO_2_ into C_4_ acids in the mesophyll via phosphoenolpyruvate carboxylase (PEPC) which diffuse to and are decarboxylated in the bundle sheaths allowing CO_2_ to be concentrated there (Fig. 2; von Caemmerer and Furbank 2003).

At the time of this meeting, some progress had already been made to express several maize photosynthetic genes in rice leaves with the goal to produce C_4_ photosynthesis in a single cell, where carboxylation by PEPC and decarboxylation by NADP-malic enzyme (NADP-ME) both occurred in mesophyll cells but partitioned between the cytosol and chloroplast (Miyao et al. 2011; Miyao 2003). While it was unknown whether such a small spatial separation of the “CO_2_ pump” from Rubisco would be sufficient for the mechanism to function efficiently, this approach did not require the challenges of anatomical specialization (Miyao et al. 2011). Initial promising reports pointed toward yield and growth increases with a partial pathway installed (Ku et al. 2000) later proving difficult to replicate and no functional C_4_ photosynthetic flux was ever demonstrated (Fukayama et al. 2003).

In 2006, at a follow up meeting at IRRI, the C_4_ Rice Consortium was born with a group of 16 laboratories in 11 countries resolving to take on the challenge of engineering C_4_ rice. The proceedings of this meeting (Sheehy et al. 2007) laid out the ground work for what was to become the most challenging crop engineering project ever attempted; what we would now call a major challenge in synthetic biology. Over the following 2 years, project plans were painstakingly prepared, timelines of ≥ 15 years for a prototype proposed and in 2008 a group of scientists representing the consortium presented the project to the Bill and Melinda Gates Foundation in Seattle. The backdrop to this presentation was what is now known as the global food crisis of 2008; a major deficit in global food supplies, the doubling of rice grain prices in less than a year, and severe shortages of wheat grain resulting in starvation and food riots in Cairo (FAO 2009). A perfect storm to support the funding of what many researchers called an “Apollo project”; as difficult as putting a man on the moon.

## Building the tools to construct the prototype

C_4_ photosynthesis has evolved independently more than 65 times in nature (Sage et al. 2012). This has been achieved using three major biochemical variants of the pathway; NADP-malic enzyme (NADP-ME), NAD-ME malic (NAD-ME) and the Pepcarboxykinase (PCK) type (Furbank 2011), so named for the bundle sheath enzyme used to decarboxylate the C_4_ acid produced in the mesophyll cells to release CO_2_. Most cultivated C_4_ crops (such as maize, sorghum and sugarcane) utilize NADP-ME as their primary decarboxylase, leading the consortium to choose maize as the model for the C_4_ Rice prototype. Figure 2 shows the basic requirements of a C_4_ mechanism in rice including the key enzymes and chloroplast transport steps. Previous reviews have dealt in depth with the biochemical and anatomical specialization in C_4_ plants believed to have evolved to support the photosynthetic mechanism (von Caemmerer et al. 2012; Langdale 2011; Furbank 2011; Hibberd et al. 2008; Wang et al. 2017). These include reduced vein spacing with Kranz anatomy/photosynthetic functionalization of the bundle sheath (Wang et al. 2017; Langdale 2011), more than 100-fold increases in expression of key photosynthetic enzymes, and appropriate cell preferential expression of proteins in the bundle sheath or mesophyll (Ermakova et al. 2020). Here we will address progress and challenges in building tools to address two key aspects of building the C_4_ rice prototype; high level expression of pathway enzymes and appropriate cell preferential expression in mesophyll or bundle sheath compartments of rice (Ermakova et al. 2020).

## Boosting C_4_ enzyme levels in rice; how much is enough?

An aspirational goal in C_4_ rice engineering is to reach maize levels of expression and/or activity of C_4_ enzymes preferentially expressed in the correct cell types, in a rice genotype with maize leaf anatomy. Although both maize and sorghum bicolor have exceptional high photosynthetic rates and enzyme activities, other C_4_ NADP-ME species such as *Setaria viridis* demonstrate that functional NADP-ME C_4_ photosynthesis can be achieved with a diverse range of enzyme activities (Sonawane et al. 2017; Osborn et al. 2017), mirroring the phylogenetic diversity of biochemical solutions observed across C_4_ species.

In addition to being guided by natural diversity in C_4_ pathways, we can also learn from previous work in transgenic C_4_ plants where levels of key enzymes were “titrated out” using gene suppression, as to the need to reach this aspirational goal in order to support adequate C_4_ photosynthetic fluxes in rice (Furbank et al. 1997). It can be seen in Fig. 2 that five C_4_ cycle enzymes support a minimal pathway (carbonic anhydrase (CA), PEP carboxylase (PEPC), malate dehydrogenase (MDH), NADP-malic enzyme (NADP-ME), and pyruvate phosphate dikinase (PPDK)). These enzymes have all been expressed in rice using a single construct but the amount of protein and enzyme activities need to be augmented (Ermakova et al. 2021). In Table 1 we have collated results from previous transgenic studies that have used antisense or RNAi technology to reduce the protein content of the C_4_ enzymes. It summarizes the affect a 50% reduction in enzyme content would have on photosynthetic rate. Three studies have confirmed that there is ample carbonic anhydrase activity such that a 50% reduction results in a very small decrease in photosynthetic rate. Studies in *Amaranthus edulis* and *Setaria viridis* show that 45% PEPC activity leads to 21% reduction in rate highlighting the importance of this enzyme. Reduction in, (PPDK) by 45% also leads to a 17% reduction. Reduction in MDH activity by half does not reduce photosynthetic rate and similarly, reduction of malic enzyme activity by 50% results in only a 6% reduction in photosynthetic rate. This is encouraging and suggests that less than full maize enzyme activity levels may be able to produce a functional C_4_ rice. Rubisco located in the bundle sheath can exert the strongest control although it is often co-limited by electron capacity (Furbank et al. 1996; Siebke et al. 1997). Balancing the amount of Rubisco with the C_4_ enzyme actives will be an essential fine tuning to ensure the efficiency of the pathway (von Caemmerer et al. 1997a, b).

**Table 1:** Comparison of photosynthetic rates at 100 and approximately 50% of protein content of various C4 photosynthetic enzymes

Enzyme	Species	CO_2_ assimilation rate at 100% (µmol m^−2^ s^−1^)	CO_2_ assimilation rate at 50% (µmol m^−2^ s^−1^)	percent reduction in rate	Gas exchange conditions	Publications
Carbonic anhydrase, CA	*Flaveria bidentis*	32.4	33.7	0%	25 °C, 1500 µmol quanta m^−2^ s^−1^ and CO_2_ at 400 µbar, 21% O_2_	(von Caemmerer et al. 2004)
Carbonic anhydrase, CA	*Setaria viridis*	22.5 30.0	21.7 (54%) 29.2 (30%)	3.6% 2.6%	25 °C, 1500 µmol quanta m^−2^ s^−1^ and CO_2_ at 400 µbar, 21 and 2% O_2_	(Osborn et al. 2017)
Carbonic anhydrase, CA	*Zea mays*	23.1	21.2 (50–97%)	8%	25 °C, 1000 µmol quanta m^−2^ s^−1^ and CO_2_ at 370 µbar, 21 O_2_	(Studer et al. 2014)
PEP carboxylase, PEPC	*Amaranthus edulis*	40.9	32.1 at 42%	22%	30 °C, 1500 µmol quanta m^−2^ s^−1^ and CO_2_ at 400 µbar, 5% O_2_	(Cousins et al. 2007)
PEPC	*Setaria viridis*	31.6	32 at 45%	21%	25 °C, 2000 µmol quanta m^−2^ s^−1^ and CO_2_ at 360 µbar, 21% O_2_	Serano Romero 2020 PhD Washington State University
Malate dehydrogenase, MDH	*Flaveria bidentis*	37	37	0%	25 °C, 1200 µmol quanta m^−2^ s^−1^ and CO_2_ at 400 µbar, 21% O_2_	(Furbank et al. 1997; Trevanion et al. 1997)
Pyruvate phiosphatedikinase, PPDK	*Flaveria bidentis*	37	45	17%	?	(Furbank et al. 1997)
NADP-Malic enzyme, ME	*Flaveria bidentis*	35	33	6%	25 °C, 1500 µmol quanta m^−2^ s^−1^ and CO_2_ at 400 µbar, 21% O_2_	(Pengelly et al. 2012)
Rubisco	*Flaveria bidentis*	32	28	12%	25 °C, 2000 µmol quanta m^−2^ s^−1^ and CO_2_ at 350 µl/l, 21% O_2_	(Furbank et al. 1996)
Rubisco	*Flaveria bidentis*	37	19	48%	25 °C, 1500 µmol quanta m^−2^ s^−1^ and CO_2_ at 360 µl/l, 21% O_2_	(Siebke et al. 1997, Fig. 2)

**Fig. 3:**
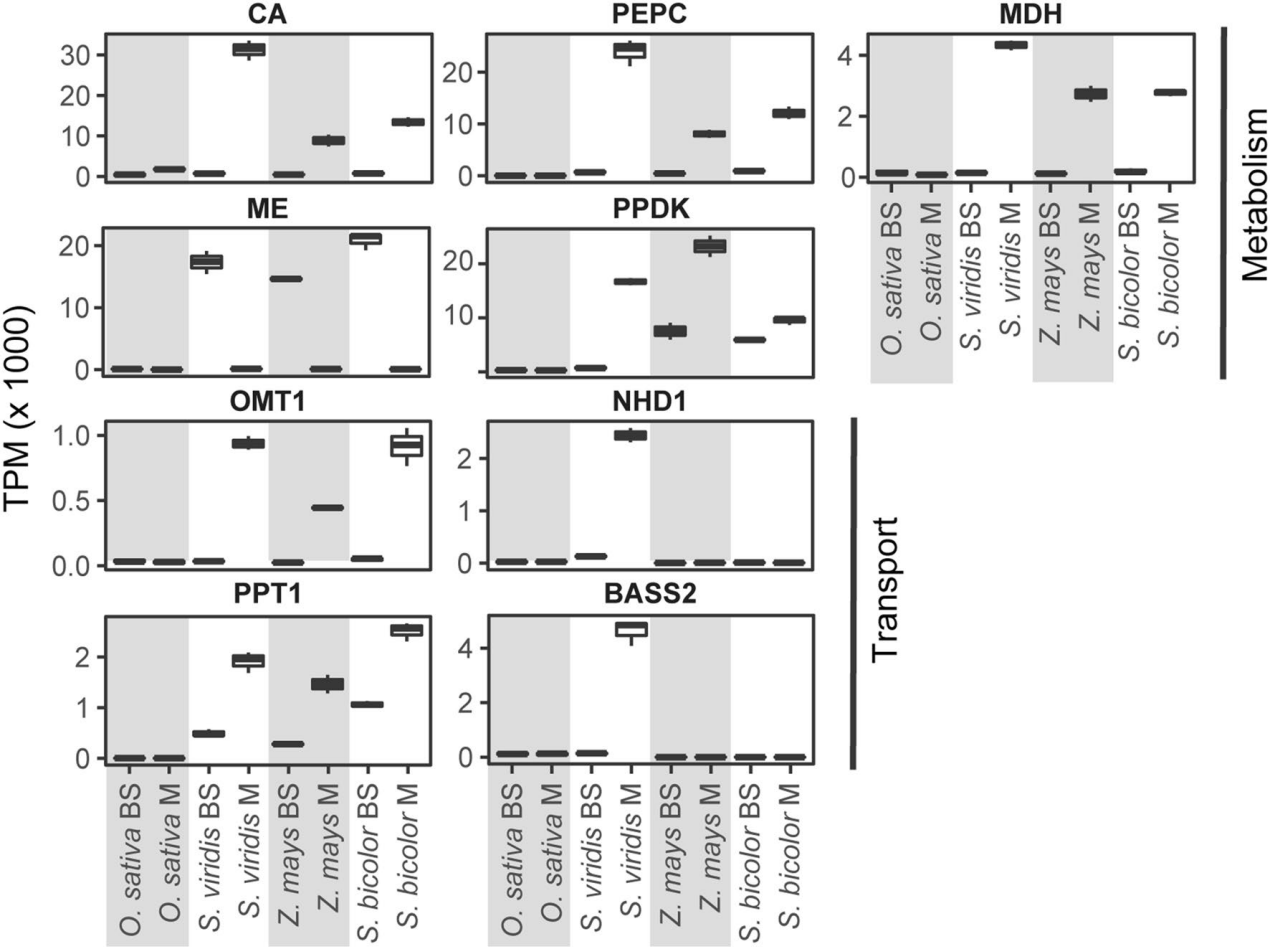
Transcript abundance of genes encoding enzymes and transporters of the C_4_ cycle. Bundle sheath and mesophyll cell transcript abundance for *Oryza sativa, Setaria viridis*, *Zea mays* and *Sorghum bicolor*. All abundance estimates are provided as transcripts per million (TPM). For abbreviations of gene names see Fig. 2

The concentration of CO_2_ around Rubisco in the bundle sheath cells of C_4_ plants is dependent on unidirectional flow of metabolites through the C_4_ pathway (Fig. 2). Achieving this unidirectional flow is critically dependent on tight cell type specific regulation of enzyme and transporter activity to limit futile cycling of C_4_ cycle metabolites. Although there are multiple ways in which cell type specific activity could be achieved (e.g. through a combination of transcriptional and/or post-translational processes), analysis of cell type specific transcriptomes revealed that regulation at the level of gene transcription has been the primary way in which cell type specificity has been achieved in different C_4_ species (John et al. 2014; Emms et al. 2016; Chang et al. 2012). The genes encoding the enzymes and transporters of the C_4_ cycle show extreme differences in transcript abundance between bundle sheath and mesophyll cells (Fig. 3), while the orthologous genes in rice show little (Fig. 3; Hua et al. 2021). Thus, the aim of the C_4_ rice project has been to achieve high levels of cell-type specific enzyme activity through the use of cell type specific promoters to drive expression of exogenous transgenes.

## Synthetic biology accelerates progress

**Fig. 4:**
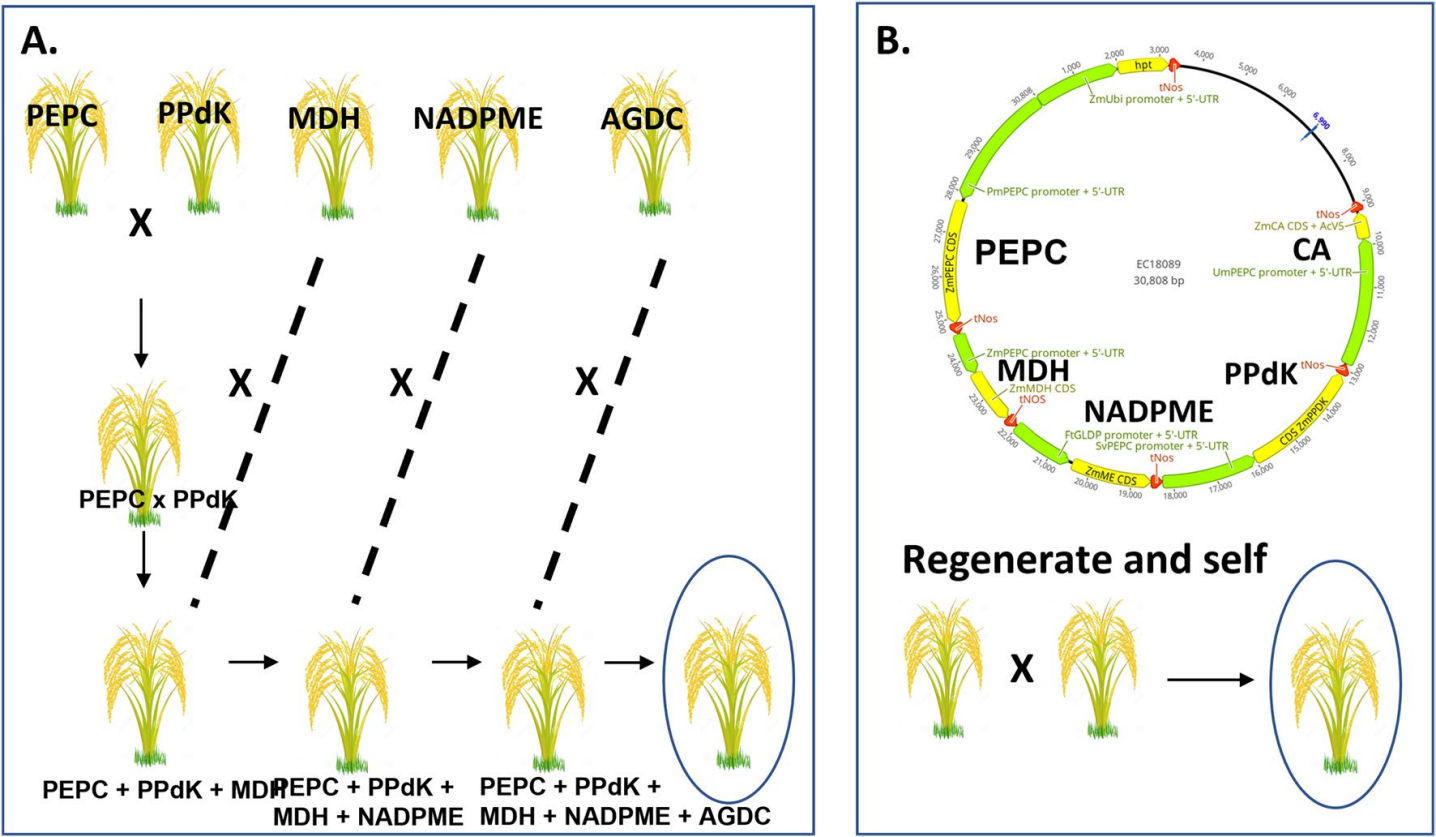
Schematic of a traditional crossing strategy to stack 5 C_4_ pathway related genes in rice similar to that used in Lin et al (2020) compared to the Golden Gate assembly method used in Ermakova et al (2020) for 5 genes using a single gene construct. The former process requires approximately 6 years of crossing and large scale genotyping due to the necessity to obtain individual lines for crossing expressing each enzymes at desired levels and homozygosity for all transgenes which will have inserted at different loci. In contrast single gene insertion means that once lines expressing all gens on the t-DNA at high levels have been obtained, they can be selfed to produce homozygous progeny in less than 1 year. Abbreviations as for Fig. 2. AGDC signifies transgenic lines where glycine decarboxylase expression has been suppressed in the mesophyll compartment

A research project which has been working toward a single goal for more than a decade is a rarity in plant science. In the course of 13 years of work, advances in synthetic biology have revolutionized pathway engineering in plants. Hierarchical Golden Gate/MoClo or Golden Braid cloning (Engler et al. 2014; Andreou and Nakayama 2018), coupled with affordable gene synthesis means that rather than creating single gene transgenic plants and crossing, the tools are now available to build gene constructs containing many genes of interest and install them in a single step (see Fig. 4 and Ermakova et al. 2020). This means that genes of interest are inserted at a single genetic locus, can easily be tracked in subsequent crossing, and homozygous individuals can be generated without many years of successive crossing and screening for plants homozygous in all transgenic insertions (Lin et al. 2020; Fig. 4). Furthermore, the parts required for gene construction can be assembled in a “toolbox” for later use with other genes of interest or refining of the approach swapping promoters, introns, untranslated and coding regions. The transgene expression challenges outlined above concerning high level and cell preferential expression are also addressed with new synthetic biology tools.

Cell specific expression of C_4_ proteins in rice has been a challenge due to the paucity of promoters known to express in the bundle sheath compartment (Ermakova et al. 2020). Until recently, a single plant promoter was available which showed bundle sheath cell preferential expression in rice; the *Zoysia japonica* PCK promoter (Nomura et al. 2005). It can be seen from Fig. 4 that several genes are required to be expressed in the bundle sheath compartment, requiring this single promoter to be re-used. There is considerable evidence in this project and elsewhere that repeating a promoter sequence in transgenes commonly results in problems with cloning due to recombination deletion or with *in planta* methylation and inactivation (Wassenegger 2002).

**Fig. 5:**
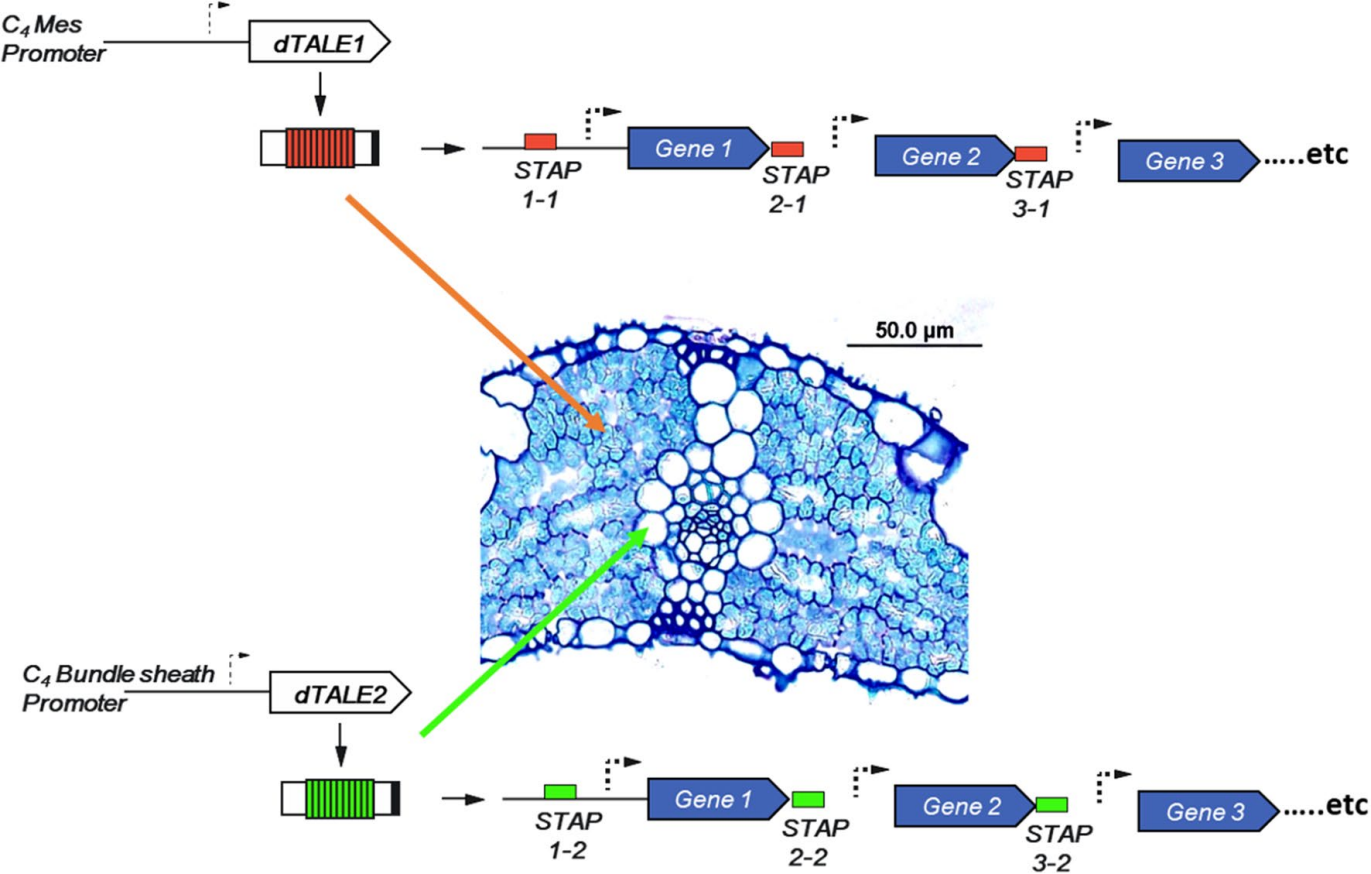
Cell specific multiplexing strategy using synthetic promoters and transcriptional activators (Danila et al 2022). Two separate dTALE transitional activators are used, each capable of activating as set of STAP synthetic promoters. In one case (dTALE1), a mesophyll promoter is used to express the transitional activator only in this compartment hence activating expression of the multiplexed transgene set in this cell type only. In the other case (dTALE2), a bundle sheath specific promoter is used to provide activation of expression in a similar way. This approach avoids the re-use of promoter sequences and the necessity to have several plant promoters specific to the target cell type

We recently demonstrated that the synthetic transcription activator-like effector (dTALE)/synthetic TALE-activated promoter (STAP) system (Brückner et al. 2015; Danila et al. 2022) provides a potential solution to the problems outlined above (Fig. 5). This system, adapted from the plant immune response to bacterial infection (Bogdanove et al. 2010), allows multiplexing of several genes on a single construct where each gene of interest is driven by a different STAP, but all STAPs are activated by a single trans activating factor or dTALE (Schreiber and Tissier 2017). In the case of C_4_ engineering, this means that a single cell preferential plant promoter can be used to drive a dTALE which activates a suite of STAPs, each driving a gene of interest in the cellular compartment where the dTALE is expressed (Fig. 5). An added advantage of this system is that it has been observed that the dTALE-STAP system can substantially amplify the strength of a weak promoter (Danila et al. 2022), potentially also addressing the problem of sufficient high-level expression in the correct cell type in the C_4_ rice prototype.

## Future challenges

The synthetic promoter system described above can potentially address the high-level expression in the correct compartment of the C_4_ enzymes in the basic pathway. “Maize levels” of every enzyme and transporter may be unnecessary to achieve appropriate flux as discussed above (Table 1). However, estimating flux through individual steps of the pathway is challenging. While extractable activity may be high, *in vivo* activity could be limited by inappropriate regulation or in fact substrate supply (Fukayama et al. 2003). The success of engineering a complete pathway can only really be judged by measurements of flux, such as via labelling into C_4_ acids and subsequent release and refixation of this carbon into the C_3_ cycle by Rubisco (Ermakova et al. 2021). If flux is low, trouble shooting of components in a complex prototype which includes multiple enzymes and transporters is challenging since localization of labelled metabolites in particular cell types or subcellular compartments is extremely difficult. Even if moderate fluxes are achieved, in the absence of the appropriate Kranz anatomy, the C_4_ pathway can likely only operate in cells adjacent to the bundle sheath cells (Ermakova et al. 2020). Vascular bundles in rice are separated by 6–9 mesophyll cells, all carrying out C_3_ photosynthesis, while only 2 mesophyll cells, devoid of Rubisco, are present between bundle sheath cells in the majority of C_4_ leaves (Ermakova et al. 2020; Sedelnikova et al. 2018). While modelling predicts measurable physiological impacts on gas exchange if maize levels of flux were achieved around the veins in rice (Ermakova et al. 2020), measuring the passage of label through C_4_ acids and then into 3-phosphoglycerate will be challenging to detect against the large background of labelled carbon appearing in 3-C compounds by direct fixation via Rubisco in mesophyll cells. For these technical reasons alone, it is desirable to combine close vein spacing and/or low Rubisco in the mesophyll tissues in subsequent prototypes. While some progress has been made on photosynthetic functionalization of the bundle sheath cells in rice (Wang et al. 2017; Ermakova et al. 2020), a major challenge remains; the discovery of a complete set of genetic switches required for the transition from C_3_ to C_4_ leaf vein spacing which remain elusive (Sedelnikova et al. 2018).

## Concluding comments

The C_4_ Rice project is progressing on several fronts. While there remain a number of unanswered questions around transport of metabolites and genes controlling vein spacing and anatomy (Wang et al. 2016; Ermakova et al. 2020), modelling suggests that even without a full complement of anatomical specialisation, we can achieve a boost in photosynthesis and yield (Ermakova et al. 2020). The rate of technological progress in plant pathway engineering and synthetic biology since the genesis of the project provides hope that we can quickly deploy scientific discoveries into our prototype, even fine tuning with rapid advances in crop gene editing, to assure success.
